# Rheumatoid arthritis: travelling biological era a Romanian X–ray population

**Published:** 2009-11-25

**Authors:** C Mogosan, V Stoica, C Mihai, L Macovei, I Ancuta, C Ciofu, F Stefanescu, M Bojinca, A Martin, M Milicescu, V Crisan, M Banciu, S Suteanu

**Affiliations:** * ‘Carol Davila’ University of Medicine and Pharmacy Bucharest, ‘Dr. Ion Cantacuzino’ Hospital, Department of Internal Medicine and Rheumatology Romania; ** Timisoara Municipal Hospital – Ambulatory of Rheumatology Romania; *** Rehabilitation Clinic – TimisoaraRomania

**Keywords:** rheumatoid arthritis, biologic and non–biologic DMARD, disability, social, utility

## Abstract

**Background**: rheumatoid arthritis (RA) is associated with loss of overall functionality of the locomotion system and it is connected with substantial economic losses.

**Objective**: to describe the clinical characteristics and healthcare resource utilization characteristics and to analyze the correlations in a cross–sectional sample of 206 patients in Romania.

**Method**: RA cases have been enrolled from southern and western part of the country, covering a surface of 23 counties.

**Results**: particularly in the literature data, Romanian RA patients become work disabled at 5.65 ± 5.99 years old after the diagnosis. At cohort level, retirement in the first year after RA diagnosis is of 22.9%. From those, 13% were treated with biologic DMARDs; those on non–biologic DMARDs were 28.6%. In oral therapy group the most prescribed drug is lefunomide (61.2%). RA has an important impact on pain, function and utility, influenced by social factors. Patients' follow up is often based on hospitalization.

**Conclusion**: currently, when the clinician may choose for one certain therapy or another, the social influence is still overwhelming at all the evaluation levels in RA patients, as well as at economic impact.

## Background

At the beginning of the third millennium, starting with getting thoroughly into the molecular mechanisms responsible for the synovitis initiation in rheumatoid arthritis (RA), medical research has reached new therapy forms, through the biological agents. After the non–biologic DMARDs (disease modifying antirheumatic drugs) era, the introduction of biological agents in the current medical practice has revolutionized the Rheumatology field. Recently, the RA evolution was described as a ‘potentially reversible/treatable physical disability’ [1]. Parallel with this new therapy introduction, many clinical studies have shown its evidence based on short term and long term efficacy, as well as tolerability [5].

Treatment with biologic DMARDs is expensive. However, the better reduction of disease activity and effect on the retirement of a long–term physical function might be cost–saving to the community, because disease improvement might lead to the improvement of the quality of life but also to improved utilization of health resources (such as hospitalizations) and reduced sick leaving and work retirements. In International cost– utility analyses, it has already been shown that the extra costs to achieve the extra benefits are acceptable with cost– utility ratios falling between 50,000–60,000 USD/ 1 QALY [5]. Is this acceptable in Romania? As long as these kinds of studies have not been performed in our country, the question still needs an answer. However, these studies were performed in Europe and North–America and cost–effectiveness analyses cannot simply be transferred to other countries which have a different healthcare and cost system. In developing countries, along with Romania, the society has not enough power to entirely cover the payment mechanisms for all the patients who would theoretically have indication for biologics. As a consequence, a centralized settlement program was developed, according to a nationally validated protocol. Based on the parameters assessment, the access to biologics is then decided. 

Considering these actualities how does the RA population in Romania looks like? What are the rheumatologists prescribing? What is the prescription trend and which are the factors that influence physicians to choose between the treatment options? Costs, benefits? What is the report between the therapy forms? These are only a few questions which need answers in order to expand the RA picture to other geographical, social and economical areas. These data might be the start for formal cost–effectiveness analyses.

### Rheumatoid arthritis: an up to date

Rheumatoid arthritis (RA) is a systemic chronic inflammatory disease, with fluctuant evolution and unpredictable prognosis [8]; it leads to severe decline in functional status and quality of life and increases morbidity and mortality [7]. 

RA induces considerable socio–economic effects [2, 10– 13]. It is known that after ten years of disease evolution, roughly half of the patients are work disabled; this brings the loss of productivity in the foreground of the RA economic impact [2, 9, 10, 14, 15].

The major therapeutically goal in RA is to interfere with the disease pathogenic paths. Stopping the joint destruction process would maintain the quality of life, through the prevention of physical disability and premature death.

Pharmacological treatment represents the main option. The group of non–biologic remission agents, generically called DMARDs, consists of: methotrexate (MTX), sulphasalasine (SSZ), leflunomide (LFL), gold salts, hydroxiclorochine, D– penicillamine, cyclophosfamide, azathioprine, cyclosporine A. MTX is currently the most used remissive agent, being therapeutically considered the ‘gold–standard’ [3].

The biologics introduction opened new perspectives at a pathogenically level (confirming the implications of the immunity elements) and at a clinical practice level, offering the alternative of the remission induction for non responders to non–biologic DMARDs. 

In Romania, the biological therapy uses anti TNF–alpha (alpha tumor necrosis factor) monoclonal antibody (Infliximab) and soluble receptors for TNF–alpha (Etanercept, Adalimumab) and anti CD20 antibodies (from B lymphocytes surface) (Rituximab). Chronologically, the first one introduced, having RA as indication was Infliximab, in 2000, followed by Etanercept, during 2004 (indicated in juvenile idiopathic arthritis and starting with 2005 in adult RA as well); during 2005 Adalimumab was also introduced. The latest biologic agent adopted in our country for clinical use is Rituximab, in 2008.

From the National Health Insurance House database, in The National Committee for the Biological Therapy Approval for RA Patients, during the first trimester of 2006, a number of 1074 patients were on biologics, in the fourth trimester of 2007 the total reaching 1500 patients, and in the same trimester of 2008 the total number of patients was 2143 (Table 1). 

**Table 1:** Rheumatoid arthritis on biological therapy in Romania

Rheumatoid arthritis on biological therapy in Romania
	Total	Infliximab	Etanercept	Adalimumab	Rituximab
Trimester 1–Year 2006	1074	954	117	3	
Trimester 4–Year 2007	1500	840	455	205	
Trimester 4–Year 2008	2143	655	902	396	190

### European Health Systems

Health systems are constantly changing. There are three main healthcare systems in Europe: ‘The National Healthcare System’, ‘The Social Health Insurances System’– Bismark and ‘The Centralized Healthcare System’– Semashko. The major differences between these are responsible for the consequences of medical practice.

NHS was at first introduced in England but nowadays it can be also found in Denmark, Italy, Finland, Ireland, Norway, Sweden, Greece, Portugal and Spain. The system is financed through general taxes, in controlled by the government and has both a state budget and a private sector. All citizens have free access to the system, the coverage is general and the state authorities manage the system. In certain cases, the patients pay a part of the cost for some medical services. Its major disadvantage consists of long waiting lists for certain medical services and a high level of bureaucracy [6].

The Social Health Insurances System is the most used one and it is based on compiling the main elements of the social and medical insurances. This system operates in Germany, Austria, Belgium, Switzerland, France, Luxembourg and Netherlands. Even if the system offers a broad coverage, a certain part of the population remains outside the coverage area of the medical services. The system financing is based on the compulsory contributions of the employers and employees [6].

The Centralized Healthcare System, introduced in Russia was typical for the Central and Eastern European countries, which are now going through a transition process to the market economy. The state had full control over the production factors and health facilities and services. The doctors were state clerks and there was no private sector. The medical assistance was free for everyone employed oversized personnel and hospitals; it had no competition and it lacked performance [6].

Just like other former socialist countries, Romania organized the national healthcare system according to Semashko Russian model, based on free access to medical services for every citizen. Despite the fact that after the communism fell and reforms in healthcare system tried to get it closer to the German model, our system still fights with true weaknesses: little health expenditure as percentage in the GDP/capita (the current allocation is of only 3.2%, compared to the necessary of 8% from GDP); centralized allocation of resources; **overrated hospital services**; lack of professional medical equipment and drugs; inequity in medical services delivery across the regions of the country [6].

Given this picture with ambiguous borders, what is really happening with RA patients who go beyond non–biologic DMARDs therapy phase being labeled as non– responsive?

## Paper objective

Having designed an observational cross sectional cohort study of cost–effectiveness of the biological treatment compared to classical DMARDs, with a follow up period of 12 months, in this paper we proposed to describe the clinical characteristics and healthcare resource utilization characteristics and to analyze the correlations between a cross–sectional sample of 206 RA patients, at baseline (December 2007). 

## Patients and methods

The lack of a National RA Register, as well as a National RA Database has imposed a different sample enrollment method. We used two sources. Some of the cases are represented by patients from the Internal Medicine and Rheumatology Department of ‘Dr. Ion Cantacuzino’ Hospital in Bucharest, during the year 2007. They have been drawn out chronologically, according to their time presentation, from the hospital electronic database. Inclusion criteria consisted of RA diagnosis and exclusion criteria, the presence of any malignancy. Through the collaboration with other rheumatologists within the country, other cases have been enrolled from the ambulatory care, in order to cover a larger territorial area: randomly, from their patients' lists, based on the same inclusion and exclusion criteria. The RA diagnosis was established by each specialist (rheumatologist) for each patient apart.

The patients – initially 480, recorded with names and addresses – were invited, through a post consent letter, to attend a scientific research. Three series of self report interviews were collected by post mail (at our address, written on the enclosed stamped envelope). The collected interval time was of six months, as following: 0 – 6 – 12 months. The first interview was conducted during November – December 2007, the second one during May – June 2008 and the last one during November –December 2008. Following the first approach, from the initially 480 cases, we collected 206 responders' envelopes (response rate being roughly 50%). These cases were considered eligible for the study; the second and third mail approach was conducted only for these last patients. 

Each serial evaluation consisted of three different questionnaires: an original one, Health Assessment Questionnaire (HAQ) Disability and Discomfort Scales – simple translation, not being validated in our country yet –and EUROQOL EQ–5D, Romanian version (having the original authors' consent for using it). 

The collected data (all self reports) were distributed on the following interest categories:

1. demographic (date of birth, age, sex, ethnic origin, marital status, environment of habitat, level of training, average income per month, professional status);
2. co–morbidities: the categories of patients with high blood pressure (HBP), diabetes mellitus (DM), chronic hepatitis (CH), coronary heart disease (CHD), gastro–duodenal ulcer (GDU) – indicating episodes of digestive bleeding, renal failure (RF), asthma (A), osteoporosis (OP), osteoarthritis (OA), thyroid gland disease (T) and others have been noted together with arthroplasty procedures and other surgical interventions;
3. concerning the major disease (RA):
  - ***General features***: diagnosis year, current treatment and its starting time, associated medication (corticotherapy /non steroidal anti–inflammatory drugs–NSAIDs).
  - ***Functional characteristics***: HAQ score, self reporting of pain intensity, disease activity and fatigue on a visual analogue scale of 100 mm (marked only at extremes), number of disabled days for usual activities, number of persons involved in home aid to its frequency.
  - ***Quality of life characteristics***: utility (EQ–5D) and EQ–domains components (RA impact on mobility, self care, usual activities, pain/discomfort, anxiety/ depression), self reporting of the health quality on a visual analogue scale of 100 mm (feeling thermometer), marked each millimeter.
  - ***Features of the economic impact*** with a time frame of 6 months: the number of sick leave days and hospitalization days, frequency for sick leave and hospitalizations, number of medical visits to the primary care and to rheumatologist, medical system appeals (regardless of the specialty), laboratory checks, number of X–rays, and CT/MRI examination, reporting on rehabilitation frequency, the patient's monthly contribution (own pocket expenses) to the treatment.

### Data analyses

**Fig 1:**
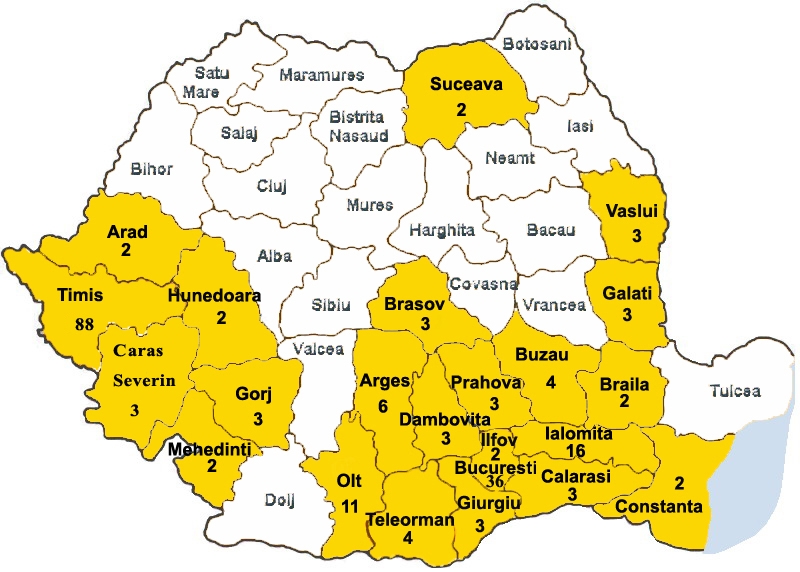
Cohort territorial distribution

**Fig 2:**
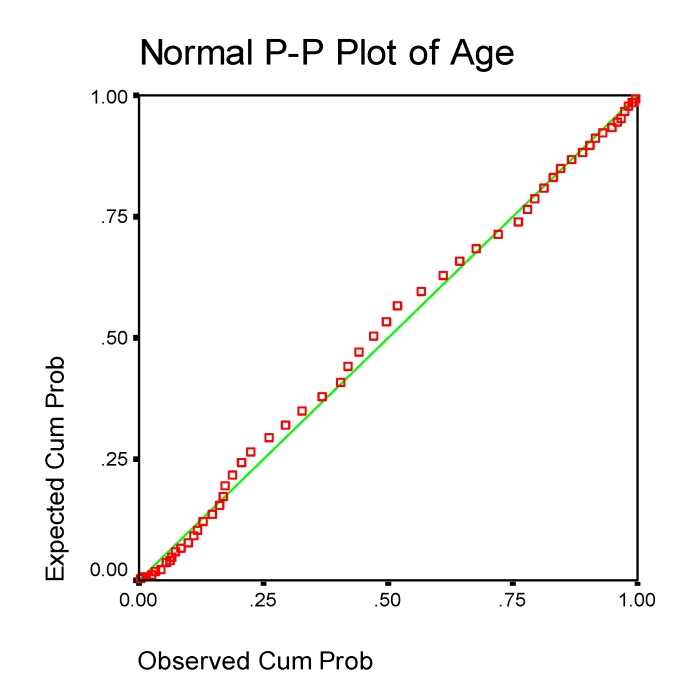
P–P plot for age (n=206)

Geographically, the sample (n = 206) covers 23 counties, from the Southern and Western part of the country (Fig 1). The large territorial distribution of the cases as well as the normality statistical sample (Fig 2), determined us to estimate that the sample is representative of the entire population suffering of RA, in our country.

The data have been analyzed in the program SPSS 10; we used ANOVA, two independent samples T test – for the continuous variables, Chi–Square, Kruskall Wallis and Man Whitney tests– for non–continuous variables, bivariate correlations (Pearson, Spearman coefficients).

The sample has been subdivided according to the therapy, as it follows: group treated with oral agents (non-biologic DMARDs monotherapy and combinations = Group A) and biological agents group (biologic DMARDs = Group B). Seven cases have been excluded from the review: five without remission therapy (the size of the subgroup being too small to be analyzed compared to the other), and two other cases that have not responded to questions regarding the medication and could not be allotted to any group. 

## Results, discussions, conclusions

Sample and subgroups features, spread over the study categories at inclusion are summarized in Table 2, Table 3, Table 4, Table 5, Table 6, and Table 7.

Results are given in average± DS for continuous variables and in percentages for non–continuous variables; a + b = 199 (7 cases have been excluded after splitting the sample into therapeutic groups); group A= non–biologic DMARDs; group B= biologic DMARDs; * Level of significance alpha: p<0,05; NS=non statistically significant 

**Table 2:** Demographic characteristics

Characteristic	Sample n = 206	Group A^a^ n = 129	Group B^b^ n = 70	p value Group A versus B
Age at inclusion (years)	54.90 ± 12.67	56.76 ± 12,25	51,84 ± 11,82	0,007*
Women	86,4%	88,4%	84,3%	NS
Urban	66%	64,3%	67,1%	NS
Married	75,7%	79,8%	71,4%	NS
Ethnicity (Romanian)	93,7%	93%	95,7%	NS
Work active	29,1%	29,5%	30%	NS
Retired	69,4%	69%	70%	NS
Unemployed	1,5%	1,6%	–	NS
Not school education	1%	1,6%	–	NS
Primary education level	48,5%	49,6%	48,6%	NS
Medium education level	36,9%	37,2%	34,3%	NS
Superior education level	13,6%	11,6%	17,1%	NS
Monthly income				
<500 lei	61%	60,5%	62,9%	NS
500 –1000 lei	29,3%	31%	25,7%	NS
1000 – 1500 lei	7,8%	7%	10%	NS
> 1500 lei	2%	1,6%	1,4%	NS

Although the patient's age in group B is significantly lower (Table 2), this difference is not the notable one in the working activity status and income. It figures a RA population with an average age of 54.90 ± 12.67 years old, which is theoretically part of the working active category. Practically, however, two thirds are retired, most cases have low monthly income (<1000 lei/month, to 90.3%), and approximately half of them have completed only primary education (it seems we are dealing with a RA population of young people, poor and elementary trained?). In this framework, between the level of education and the monthly income, there is a homogeneous significant positive correlation in both groups (ρs = 0.645, p < 0.01). These issues outline the social conditions in the demographic characteristics background.

**N.B.** Results are given in **percentages** for non–continuous variables; a + b = 199 (7 cases have been excluded after splitting the sample into therapeutic groups); group A= non–biologic DMARDs; group B= biologic DMARDs; * Level of significance alpha: p<0,05; NS=non statistically significant 

**Table 3:** Associated RA morbidities: characteristics

Characteristic	Sample n = 206	Group A^a^ n = 129	Group B^b^ n = 70	p value Group A versus B
**Total no. comorbidities**				0,007*
0	16,7%	15,5%	17,6%	
1	18,1%	19,4%	16,2%	
2	19,1%	20,9%	17,6%	
3	20,6%	17,1%	23,5%	
4	12,3%	12,4%	13,2%	
5	6,4%	6,2%	7,4%	
6	4,4%	6,2%	1,5%	
7	2,5%	2,3%	2,9%	
**Groups comorbidities**				
High blood pressure	49,5%	53,5%	42,6%	NS
Osteoporosis	40,2%	40,3%	39,7%	NS
Coronary heart disease	29,9%	32,6%	23,5%	NS
Osteoarthritis	23%	24%	22,1%	NS
Gastro–duodenal ulcer	21,1%	18,6%	27,9%	NS
Diabetes mellitus	16,2%	17,1%	16,2%	NS
Thyroid gland disease	15,7%	14,7%	17,6%	NS
Renal failure	9,8%	9,3%	11,8%	NS
Asthma	7,4%	7,8%	7,4%	NS
Others	26%	27,9%	22,1%	NS
Digestive bleeding	3,5%	4,8%	1,4%	NS
**Surgery**				
Arthroplasty	3,9%	1,6%	7,1%	0,04^*^
Other surgical procedure (out of the RA context)	9,8%	9,3%	11,4%	NS

Morbidity is significantly associated with PR (Table 3). Over half of the patients (57.3%) had associated three diseases with PR, in terms of population with an average age of 54.90 ± 12.67 years old. Between them, the first three places are occupied by high blood pressure, osteoporosis and coronary heart disease; as already confirmed, cardiovascular diseases increase the mortality rate, independently. It also remarks the significantly higher arthroplasty rate in B (7.1%, compared to 1.6%; p<0.05): it refers to a history of more severe diseases for the current biologics cases.

**N.B.** Results are given in **average±DS** for continuous variables and in percentages for non–continuous variables; a + b = 199 (7 cases have been excluded after splitting the sample into therapeutic groups); c percentages include monotherapy and combinations; group A= non–biologic DMARDs; group B= biologic DMARDs; * Level of significance alpha: p<0,05; NS=non statistically significant; NA = not applicable; NSAIDs =non steroidal anti–inflammatory drugs; MTX=methotrexate; SSZ= sulphasalazine; LFL = leflunomide.

**Table 4:** General characteristics of rheumatoid arthritis

Characteristic	Sample n = 206	Group A^a^ n = 129	Group B^b^ n = 70	p value Group A versus B
**Disease duration starting diagnosis (ys) **	9,40 ʱ 8,87	8,24 ʱ 8,93	11,32 ʱ 8,30	0,01^*^
**Treatment: therapeutically groups**				
No remissive treatment	2,5%	–	–	NA
Non–biologic DMARDs monotherapy	47,1%	74,4%	–	NA
Non–biologic DMARDs combinations	16,2%	25,5%	–	NA
Biologic DMARDs plus MTX	25,5%	–	74,3%	NA
Biologic DMARDs without MTX	8,8%	–	25,7%	NA
**Treatment: DMARD preparates^c^**				
MTX	48,6%	36,4%	74,3%	NA
SSZ	19,6%	27,1%	7,1%	NA
LFL	42,2%	61,2%	10%	NA
**Treatment: biologic agents**				
Infliximab	65,7%	–	65,7%	NA
Etanercept	20%	–	20%	NA
Adalimumab	14,3%	–	14,3%	NA
Mean duration of current treatment (ys)	2,70 ± 2,64	2,71 ± 2,86	2,71 ± 2,19	NS
**Anti–inflammatory drugs**				
NSAIDs	89,2%	91,4%	88,2%	NS
Monthly NSAIDs intake				
None	10,8%	9,1%	11,9%	NS
<10 days	17%	19,0%	14,9%	NS
10 – 20 days	25,3%	26,4%	23,9%	NS
> 20 days	11,3%	8,3%	16,4%	NS
Daily	35,6%	37,2%	32,8%	NS
Corticotherapy	39%	37,8%	39,7%	NS

Average disease duration is of 9.40 ± 8.87 years old, with a significant difference between groups in favor of biologics (11.32 ± 8.30 years old). In other words, biological agents predominate in older forms of the disease at a younger category of patients. If in group A, RA age increases linearly with age (r = 0.233, p < 0.01) in group B these factors are independent, supporting a broader distribution of the cases on the age axis.

On figures (Table 4), two thirds of patients are following non–biologic DMARDs (¾ monotherapy, ¼ combinations), and one third, biological agents. Interesting is that assessing the entire sample, the most prescribed DMARD seems to be MTX (48.6%) – including here MTX prescriptions associated with biologics–while looking only in group A (non– biologic DMARDs), first place is occupied by LFL (61.2%). The explanation relies on two aspects: on the one hand, surprising RA population at approximately 10 years from the disease evolution, when most patients are beyond stage MTX, either through inefficiency (secondary resistance) or by cumulative dose over time; on the other hand, the influence role of various pharmaceutical companies in prescribing a certain drug should not be missed. What is interesting about the analyzed population is that other DMARDs preparations were not declared, which draws attention to a phase of decline in using a medication formerly overused (gold salts, hydroxycloroquine etc.), as well as to a selective promotion of products from pharmaceutical companies. In the biologics group, the ‘oldest’ drug is placed on top of the most prescribed one: Infliximab (65.7%). This feature follows the national wide distribution of TNF blockers agents in ‘The National Committee for the Biological Therapy Approval’ (Table 1), where Infiliximab was the most frequently biologic at the end of 2007. We conclude that these figures reflect a stage of plateau in the dynamic of the described parameters, as the average duration of the reported treatment is of 2.70 ± 2.64 years old.

AINS consumption is high (89%), without differences between groups. Looking at the figures, over ⅓ of the cases require daily NSAIDs (35.6%). Considering correlation with age and disease duration, NSAIDs intake increased with RA age only in group A (ρs = 0.212; p< 0.05). Supporting these data with associated pathology, the risk of adverse events, even fatal (by major cardio–vascular disorders) is amplified.

**Fig 3:**
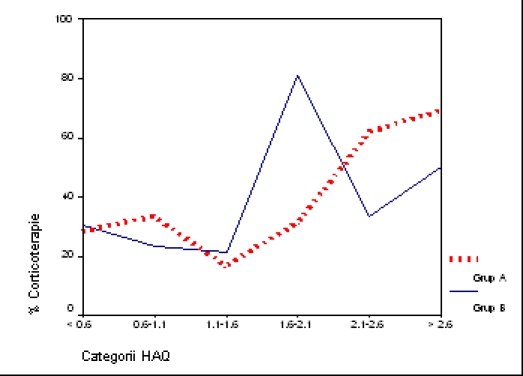
Corticotherapy in relation with HAQ categories

Corticotherapy is part of the treatment in 39% of the cases. Looking at sample level, the relationship corticotherapy –  HAQ disability categories (Fig 3), cortisone therapy is missing in 73% of the cases for HAQ categories <1.6; starting with HAQ scores > 1.6 corticotherapy is present at 59% (p <0.01). The ratios are different in subgroups. Group A describes a similar curve, except for the report reversal point starting with HAQ values of > 2.1. On the contrary, in group B cortisone therapy is present in 81% of the cases belonging to the HAQ category: 1.6–2.1; in all other intervals, the majority of the patients do not require cortisone therapy, even for HAQ values <1.6, as for those > 2.1 (75 % and 58% patients, respectively), p < 0.01.

Surprisingly, group B described a positive correlation (gs = 0.247, p < 0.05) between corticotherapy and age. It clearly appears that the oldest patients belong mostly to HAQ interval 1.1– 2.1 (meaning moderate and advanced disability), the same area recorded a peak in cortisone therapy, as well. 

At least two conclusions can be detached: in oral therapy group, ‘RA end stage’ of irreversible disability (27.2%) is frequently cortisone dependent; in this group, corticotherapy describes a linear growth in the degree of disability, in order to maintain a minimum functionality of the locomotion system. On the contrary, in biologics group cortisone prescription follows the disability intervals potentially reversible (HAQ 1.1 – 2.1), where the case density is also the highest (51.5%).

Is working activity status related to the consumption of NSAIDs and cortisone? In group B, the retired cases correlate positively with corticotherapy (ρs = 0.260, p < 0.05) and with NSAIDs intake (ρs = 0.265, p < 0.05), while the correlation is negative for the active professional cases. This reflects the former severity of the disease in group B: more severe disease (HAQ represents partly the cumulative disease activity – damage over time) reduces ability to work and makes it more likely to receive steroids and/or NSAIDs. Considering that groups have not significant differences concerning the active professional proportion, it seems that in the oral therapy group, these factors are independent. It could also reflect a former less severity of the disease in group A.

In functional terms (Table 5) the average HAQ score at baseline was of 1.29 ± 0.80. If we look to HAQ categories, household activities recorded the most severe score (36.9% of cases), followed by hygiene (17.9%).

**Fig 4:**
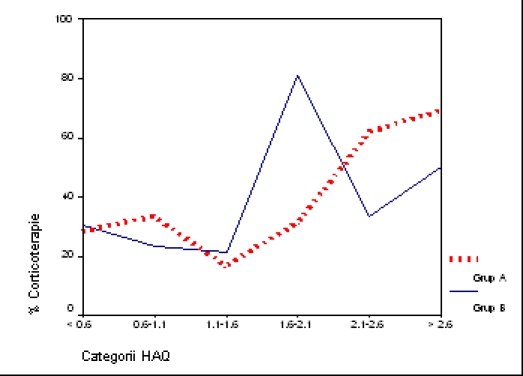
HAQ categories according to therapy groups

Considering 6 categories of severity, corresponding to a certain range of HAQ score, we mention: in group A, 27.2% of cases are severely and very severely disabled, compared to 15.7% in group B (p < 0.05). Advanced and moderate disability is observed in 51.5% of cases representing group B, compared to 27.1% of group A (p < 0.05) (Fig 4). In other words, RA terminal phases (end–stage) which have less therapeutically benefits in functional terms are mostly treated with classical agents, while the potentially reversible stages of RA disability can be found especially in the group receiving a more expensive therapy. In a society with limited health resources, this ‘selective affiliation’ of cases is predictable, considering as a therapeutic target also the socio–economic reinsertion of patients.

Age is a factor that increases the degree of disability appreciated by HAQ (r = 0.417, p < 0.01); on the contrary, less obvious correlation of disability in relation to disease duration (r = 0.251, p < 0.05). In the same polarity, we mention HAQ influence on the retired status (gs = 0.318, p < 0.01).

Using of self–quantitative VAS scale showed unexpected high scores for pain (mean 54.45 ± 24.23), disease activity (mean 55.17 ± 23.12) and fatigue (57.49 ± 24.87). Age is an inflexible characteristic, but its influence on self reporting assessments is confirmed by the positive correlation with the mentioned variables (r = 0.219, 0.259, 0.272, p < 0.01).

Although approximately ¼ of the cohort (22.8%) belongs to a functional irreversible RA phase, needed assistance in everyday life overcomes the expected level: 86.7% of patients require household help and 58.3% of them frequently report and permanently help. One single person is involved in house hold help for most of the cases (68.8%), 41.7% of patients call for auxiliary objects and 35% for aid devices, mostly for the category of usual daily activities (64.6%).

N.B. Results are given in **average± DS** for continuous variables and in percentages for non– continuous variables; a + b = 199 (7 cases have been excluded after splitting the sample into therapeutic groups); group A= non biologic DMARDs; group B= biologic DMARDs; * Level of significance alpha: p<0,05; NS=non statistically significant.

**Table 5:** RA functional characteristics

Characteristic	Sample n = 206	Group A^a^ n = 129	Group B^b^ n = 70	p value Group A versus B
HAQ score	1,29 ± 0,80	1,27 ± 0,84	1,34 ± 0,72	NS
**Severity categories (HAQ score)**				0,02 ^*^
Least Disability: < 0,6	21,4%	24,8%	14,3%	
Mild Disability: 0,6 – 1,1	19,9%	20,9%	18,6%	
Moderate Disability: 1,1 – 1,6	19,4%	14,7%	28,6%	
Advanced Disability: 1,6 – 2,1	16,5%	12,4%	22,9%	
Severe Disability: 2,1 – 2,6	14,1%	16,3%	10,0%	
Very severe Disability: > 2,6	8,7%	10,9%	5,7%	
Frequency of helping object	41,7%	39,5%	45,7%	NS
Frequency of helping device	35%	32,6%	40%	NS
Most severe HAQ score category				NS
Daily activities	36,9%	39,2%	34,4%	
Hygiene	17,9%	20,6%	14,8%	
Dressing and self–care	10,7%	9,8%	11,5%	
HAQ helping categories				NS
Daily activities	64,6%	62%	68,6%	
Grip	47,6%	48,8%	45,7%	
Reach	39,8%	40,3%	40%	
Hygiene	25,7%	22,5%	30%	
Self reporting through VAS				NS
Pain	54,45 ± 24,23	54,94 ± 24,88	54,22 ± 22,83	
Disease activity	55,17 ± 23,12	56,30 ± 23,83	53,46 ± 21,57	
Fatigue	57,49 ± 24,87	58,50 ± 25,91	56,68 ± 22,59	
Monthly lost days for usual activities	9,64 ± 9,06	9,12 ± 9,32	10,43 ± 8,55	NS
Frequency of household help	86,7%	85,7%	88,4%	NS
Rare	28,4%	28,6%	29,0%	
Frequent	32,5%	29,4%	36,2%	
Permanent	25,8%	27,7%	23,2%	
Persons needed for household help				NS
1	68,8%	66,1%	73,9%	
2	8,5%	8,1%	10,1%	
3	2%	1,6%	2,9%

Overall functional impact in daily life is materialized in about ten days lost every month because of the inability of performing household tasks (average: 9.64 ± 9.06 days). There is an important and relatively homogeneous positive correlation between lost days and the self reporting level for pain, fatigue and disease activity (r = 0.650, p < 0.01) in both groups.

The category of working active cases has a certain degree of independence at home. Both groups reveal a negative correlation of these cases with lost days for usual activities (gs = –0.255 and –0.351, p < 0.01), while in group A the help frequency is bigger (gs =– 0.307, p < 0.01); pensioners group correlation is positive (meaning an increase in non–medical direct costs).

Utility (Table 6), appreciated on a scale from 0 to 1, where 1 corresponds to perfect quality and 0 to death, is placed for the whole sample to an average of 0.417 ± 0.337. Between groups, there is a difference in favor of biologics (0.382 ± 0.347 and 0.452 ± 0.317, p = 0.1), but both figures belong to a low level. The most frequently reported score was of 0.516 and it was found in one third of cases. It is interesting that the proportion of those who reported moderate and severe problems in the utility components stratify somehow the RA impact in patient daily life. Thus, 95.5% reported pain / discomfort, 83% have problems in mobility and usual activities, 74% in self care and 69% have anxiety / depression.

The quality of health state assessed through VAS showed an average score of 47.39 ± 22.13, with a statistically significant difference between groups in favor of biologics (44.92 ± 22.34, and 51.36 ± 21, 23, p < 0.05).

N.B. Results are given in **average±DS** for continuous variables and in percentages for non– continuous variables;^**^ Percentages represent frequency of problems (moderate and severe) in mentioned category; a + b = 199 (7 cases have been excluded after splitting the sample into therapeutic groups); group A= non biologic DMARDs; group B= biologic DMARDs; ^*^ Level of significance alpha: p<0,05; NS=non statistically significant.

**Table 6:** Utility and quality of life parameters

Characteristic	Sample n = 206	Group A^a^ n = 129	Group B^b^ n = 70	p value Group A versus B
**Utility – EQ5D**	0,417 ± 0,337	0,382 ± 0,347	0,452 ± 0,317	0,1
Most frequently reported utility values				NS
0,516	32,7%	31,7%	33,3%	
0,587	10,2%	9,2%	11,6%	
Utility components^**^				
Mobility	83%	84,6%	82,9%	NS
Self care	73,9%	73%	75,7%	NS
Usual activities	83,4%	83,6%	84,3%	NS
Pain/Discomfort	95,5%	98,4%	92,9%	0,03^*^
Anxiety/Depression	69%	71,9%	65,2%	NS
EQ5D – VAS: quality of health state	47,39 ± 22.13	44,92 ± 22,34	51,36 ± 21,23	0,05^*^

In both subgroups increased disability lowers the quality of health state and utility. (group A: gs = –0.618, –0.665 (p < 0.01); gs group B = –0.707 – 0.552 (p < 0.01).

Given the social context of RA patient in Romania, we consider it appropriate to present the correlations of some social elements with quality of life components, independently of other factors directly related to RA:

- The increasing level of education is associated with health state quality and utility: r = 0.377 and r = 0.380, where p < 0.01.
- There is an association between the standard of living caused by a low monthly income and utility score, as well as quality of health state: r = 0.323 and r = 0.364, where p < 0.01.
- There is an association between the low monthly income and the severity of utility components: mobility (gs = –0.186), self care (gs =–0.302), usual activities (gs = – 0.304), pain/discomfort (gs = –0.233), anxiety/depression (ρs = –0.216), where p < 0.01.
- There is an association between inactivity (retired group) and the severity of utility components: gs = 0.224; 0.250; 0.255; 0.167; 0.220, where p < 0.01.

In order to improve the RA impact at individual, social and economic level, some supporting and stimulating measures of any working activity, tailored according to the disease functionality are required. 

Analyzing the effectiveness, strictly in terms of utility and quality of health state, the biologics class is clearly superior to classical therapy. Expanding to the level of economic impact characteristics, the differences between groups did not describe the same behavior (Table 7).

Reported to the active professional subgroup, labor productivity was evaluated by sick leave and early retirement.

Quantifying the absenteeism frequency, 20% of the patients reported sick leave in the last 6 months. There is however a significant difference in sick leave duration, in favor of group A (mean 3.58 ± 8.66 days, compared to 0.43 ± 1.08 days in group B, p < 0.05). The analysis of correlation with the hospitalization duration revealed that the two features are independent. As a result, in DMARDs group longer sick leaves is not a consequence of hospitalization, but probably of outpatient visits in the primary care or rheumatologist, the only ones who can fix sick leave in ambulatory care.

Labor productivity loss through early retirement reaches a threshold of 38.5% of cases (33.7% in group A and 46.9% in group B) at a median duration after RA diagnosis time of only 5.65 ± 5.99 years (also the comparable average between groups). What is interesting is that in the first year after RA diagnosis, 22.9% of the newly diagnosed patients are medically retired (28.6% belonging to group A and 13% to group B). Recall that literature sustains a loss of labor productivity through work incapacity of about 50% in the first 10 years after diagnosis [15].

N.B. Results are given in average± DS for continuous variables and in percentages for non-continuous variables; a + b = 199 (7 cases have been excluded after splitting the sample into therapeutic groups); ^c^: n = 60 (working active subgroup); ^d^: n = 143 (retired subgroup); ^e^: n = 55 (RA retired subgroup);  group A= non biologic DMARDs; group B= biologic DMARDs; * Level of significance alpha: p<0,05; NS=non statistically significant.

**Table 7:** RA economic impact characteristics

Characteristic	Sample n = 206	Group A^a^ n = 129	Group B^b^ n = 70	p value Group A versus B
Sick leave /6 months ^c^	20%	23,7%	14,3%	NS
Days of sick leave / 6 months ^c^	2,42 ± 7,06	3,58 ± 8,66	0,43 ± 1,08	0,03^*^
hospitalizations / 6 months	52,4%	47,2%	67,1%	0,007^*^
Days of hospitalization / 6 months	5,42 ± 7,67	4,79 ± 7,21	6,82 ± 8,52	0,08
Loss of labor productivity: RA pensioners ^d^	38,5%	33,7%	46,9%	NS
RA retirement after diagnosis time – years	5,65 ± 5,99	5,76 ± 6,81	5,52 ± 5,33	NS
RA retirement in FIRST YEAR after diagnosis ^e ^	22,9%	28,6%	13%	0,1
Rheumatologist visits /6 months				0,000^*^
6 visits	32,3%	39,2%	21,4%	
3 visits	19,4%	11,2%	35,7%	
2 visits	15,4%	18,4%	8,6%	
0 visits	3%	4,8%	–	
Primary care visits (GP) / 6 months				NS
6 visits	41,7%	46%	34,8%	
3 visits	7,5%	6,5%	8,7%	
2 visits	8,5%	6,5%	11,6%	
0 visits	15,6%	16,1%	14,5%	
Medical system appeal (global) / 3 months				NS
3 appeals	26,9%	27,1%	27,5%	
2 appeals	10,4%	7,6%	15,9%	
1 appeal	15,5%	13,6%	17,4%	
0 appeals	13,5%	17,8%	7,2%	
Specialty (regardless of rheumatology and GP)				NS
Cardiology	4,4%	4,7%	2,9%	
Gynecology	3,9%	3,1%	4,3%	
Endocrinology	2,9%	3,9%	1,4%	
Diabetology	2,4%	1,6%	4,3%	
Dermatology	2,4%	3,1%	1,4%	
Lab tests sets				0,004^*^
3 sets	26,2%	21,4%	35,7%	
2 sets	39,6%	42,9%	34,3%	
1 set	16,8%	19,8%	10%	
0 sets	1%	0,8%	–	
Xrays number				0,008^*^
>3 Xays	9,4%	12,6%	2,9%	
1 – 3 Xrays	50,7%	55,1%	45,7%	
0 Xray	39,9%	32,3%	51,4%	
CT	0,5%	0,8%	–	NS
MRI	0,5%	0,8%	–	
Own pocket expenses/ monthly				NS
<50 lei	34,7%	38,5%	26,5%	
50 – 100 lei	39,8%	36,1%	45,6%	
< 100 lei	25,5%	25,4%	27,9%	
Rehabilitation	10,8%	10,2%	11,4%	NS

What caused the patients to be declared work disabled so early? No evident correlations (factors with independent behavior) were found between the disability degree (HAQ) and the time elapsed from the diagnosis moment to the RA retirement. On the contrary, there is a strong positive correlation (r = 0.758, p < 0.01) between RA age and time elapsed from diagnosis until RA retirement. By adding age in this equation, no additional information result was found. From this perspective, RA age, not patient age, comes in the foreground of RA impact on labor productivity.

However, figures show that a significant proportion of patients are declared work disabled at less than one year from the time of diagnosis. From this perspective, RA age losses the top position in final decision on work capacity. Considering also some social factors, we found interesting associations:

- reduction of monthly income is associated with time from RA diagnosis to ill retirement (r = 0.565, where p < 0.01;
- the highest the education level is, the greater the tendency to remain active professionally results (significant positive correlation in both groups, but more expressed in group B: gs = 0.466, p < 0.01).

In conclusion, it seems that in our country the social level of RA patients plays also a major role in the loss of work productivity. This socio–economic weakness supports a vicious circle: ‘small proportion of work active population – insufficient funds allocated in the health system– great selection and low accessibility of patients to more expensive therapies, even if with superior efficiency’.

Regarding the follow up visits to the rheumatologist, ⅓ of cases reported monthly visits, the differences being statistically significant between groups (39%–group A, compared with 21% – group B, p < 0.01); 20% of cases are monitored at intervals of 2 months (11.2% group A, compared with 35.7% in group B, p < 0.01). From the entire cohort perspective, the rhythm of monitoring visits has no correlation with the disability severity (HAQ). Given this aspect and also considering the economic implications of a medical check, a question arises: what induces the rhythm of follow up? Looking within groups on disability levels, patients with more severe disabilities are monitored on monthly basis; differences occur in cases of HAQ interval 0.6 – 2.1. Thus, in group A, most cases are monitored monthly; in group B dominate 2 months visits. In terms of the drug prescription, most of non–biologic DMARDs recipes belong to the primary care network. Thus, monthly rheumatology visits for the lower categories of disability could have two interpretations: on one hand, it could support the excess use of health care departments (inside and outside hospital), but on the other hand the patients could have better function because they come to the hospital more frequently. The study design implies 1 year of follow up, so the patient's characteristics dynamic will support one of these two hypotheses (which will be reported, as well). In group B, two months follow up is probably related to the administration rhythm of infliximab. At this point, it seems that rheumatology monitoring rhythm is determined by the clinician. 

41.7% of cases appeal monthly the primary care network and 27% have monthly visits to other specialties; cardiology hold on the top. 

Regarding the lab tests monitoring, approximately ¼ cases are tested at 2 months interval, but with significant differences between groups: 35.7% in group B, compared to 21.4% in group A (p < 0.01). The biologic patient is therefore more closely monitored biologically. These data reflect physician option.

Radiological monitoring revealed that about ¼ of cases failed to X–ray control in the last 6 months, with significant differences between groups (32% group A, group B 51%, p < 0.01), while one and half of cases had from 1 to 3 radiographs.

For 75% of cases, the monthly patient contribution to the treatment is less than 100 lei, with no significant differences between groups. This level of own pocket expenses represent 10% of the average monthly income, for 90% of the patients included.

Concerning direct medical costs level, the economic impact reveals that hospitalizations rate (reported for half of the cases) is significantly higher in group B (67.1% versus 47.2%, p <0.01). Although no significant differences between groups as extent of hospitalizations (6.82 days and 4.79 days), it seems that with age increase, only patients treated with biological agents require more frequent hospitalizations and of longer duration (r = 0.529, p < 0.01), amplifying direct costs, eventually.

The frequency and duration of hospitalization is directly related to the degree of disability in group A (gs = 0.323, p < 0.01), while in group B, it is valid for the duration of hospitalization, not for its frequency (gs = 0, 329; p < 0.01).

With respect to the superior hospitalization rate in group B, there is not a discrepancy without explanation. The reason lies in the large proportion of biologics patients treated with Infliximab, which is managed only through hospital admission. 

In conclusion, in our country, the rate of hospitalizations is not only a consequence of RA relapse episodes. The current health care system services still hospitalized based, associated to a particular social context, could increase direct medical costs in cases not related to compulsory hospitalization. The claim requires evidence in support of monetary unit, providing by data analysis which will be soon reported. 
